# Epidemiology and treatment approaches in management of invasive fungal infections in hematological malignancies: Results from a single-centre study

**DOI:** 10.1371/journal.pone.0216715

**Published:** 2019-05-09

**Authors:** Nicola Stefano Fracchiolla, Mariarita Sciumè, Nicola Orofino, Francesca Guidotti, Anna Grancini, Fabrizio Cavalca, Alessandra Freyrie, Maria Cecilia Goldaniga, Dario Consonni, Veronica Mattiello, Loredana Pettine, Agostino Cortelezzi

**Affiliations:** 1 Hematology Unit, Fondazione IRCCS Ca’ Granda Ospedale Maggiore Policlinico, Milan, Italy; 2 U.O. Laboratorio di Microbiologia, Laboratorio di Analisi Chimico Cliniche e Microbiologia, Fondazione IRCCS Ca’ Granda Ospedale Maggiore Policlinico, Milan, Italy; 3 Università degli Studi di Milano, Milan, Italy; 4 Epidemiology Unit, Fondazione IRCCS Ca' Granda Ospedale Maggiore Policlinico, Milan, Italy; University of Mississippi Medical Center, UNITED STATES

## Abstract

Invasive fungal infections (IFIs) are a leading cause of morbidity and attributable mortality in oncohematologic patients. Timely diagnosis is essential but challenging. Herein we retrospectively describe 221 cases of antifungal treatments (AFT) administered in a monocentric real-life cohort of hematological malignancies. Between January 2010 and July 2017, 196 oncohematologic patients were treated with AFT at our Hematology Department. Diagnosis of IFIs was carried out according to EORTC/MSG-2008 guidelines.The most represented disease was acute myeloid leukemia (104 patients). Median age was 61 years; at fever onset 177 (80%) patients had a neutrophil count<0.5x10^9^/L. Twenty-nine (13%) patients were receiving antifungal prophylaxis (26 posaconazole, 2 fluconazole, 1 itraconazole). The incidence of AFT was 13%. Serum galactomannan antigen (GM) was positive in 20% of the tested cases, while 85% of the patients had a CT scan suggestive for IFI. Twenty-one percent of these cases had a GM positive. Sixty-five out of 196 patients (33%) showed positive culture results, in particular Candida spp. were identified in 45 isolates, while Aspergillus spp. in 16 cases. Fourteen patients presented multiple positivity. Twenty-two (10%) cases were classified as proven IFIs, 61 (28%) as probable and 81 (37%) as possible, but 57 (26%) cases could not be classified. Fifty-nine percent of the patients received single agent AFT, 37% sequential AFT, 8% a combination regimen. Liposomal-amphotericin-B was the most used AFT. IFIs attributable mortality was 20%. This epidemiologic survey underlined a persistent significant use of AFT and a high mortality rate of IFIs. We suggest that further powerful diagnostic approaches should be investigated to improve the diagnostic accuracy and potential therapeutic implication.

## Introduction

Invasive fungal infections (IFIs) are a leading cause of morbidity and attributable mortality in patients with hematologic diseases and particularly in those receiving intensive chemotherapy or undergoing hematopoietic stem cell transplant (HSCT) [1–3].

In the last years, improvement in the treatment of hematologic cancers has been paralleled by the persistence of a relevant IFIs incidence worldwide: long lasting neutropenia due to intensification of cytotoxic chemotherapy, prolonged administration of corticosteroids, increased use of allogeneic HSCT and subsequent incidence of graft-versus-host disease (GVHD), together with the widespread use of immunosuppressive agents and the improved overall survival, contributed to this phenomenon [2–5].

In hematologic patients, the most frequent invasive fungal pathogens are Candida and Aspergillus species (spp) [1–3]. In particular, the epidemiology of IFIs is now changing, with an increase in non-albicans Candida, non-fumigatus Aspergillus spp and uncommon molds, due to multiple factors, among which the improvement in diagnostic tools and the widespread antifungal prophylaxis may play a significant role [1–8]. A study based on autoptical findings confirmed that acute myelogenous leukaemia (AML) and myelodysplastic syndromes (MDS) are the hematologic malignancies more frequently associated with IFIs. Furthermore, high rates of proven invasive fungal disease has been recently noted in patients with non-Hodgkin lymphomas (NHL) in the era of target drugs [9–10].

Timely diagnosis of IFIs is essential and a prompt appropriate systemic antifungal therapy (AFT) has been demonstrated to improve outcomes [11–15]. However, IFIs clinical presentation is often difficult to differentiate from other infections, making early diagnosis complex, especially in immunocompromised patients [16–18].

European Organization for Research and Treatment of Cancer/Invasive Fungal Infections Cooperative Group and the National Institute of Allergy and Infectious Diseases Mycoses Study Group (EORTC/MSG) defined IFIs as proven, probable or possible, based on diagnostic certainty [16]. According to these definitions, proven IFI requires a microbiological and/or histopathological diagnostic method. These procedures have major limitations, represented by a low sensitivity and specificity, a long time required for analysis, and a high complication risk for bioptic procedures in hematologic patients. To facilitate an early IFI diagnosis, non-invasive diagnostic tools were introduced, including imaging with computed tomography (CT) and magnetic resonance imaging scans, serological testing [galactomannan (GM) assay for Aspergillus, serum (1,3)-β-D-glucan (BDG) antigen test] and molecular techniques (PCR-based assays) [18–21].

To determine the best antifungal therapeutic strategy remains a challenging issue in neutropenic patients with hematological diseases with suspected IFIs, and is a matter of active debate. Some Authors suggest a risk-adapted antifungal strategy for leukemic patients, considering pre- and post-treatment variables in order to act more individualized interventions [12,22]. Pre-emptive approach based on the incorporation of sensitive, non-invasive diagnostic tests (GM antigen search and CT-scanning) for high-risk neutropenic patients, has been shown to reduce the rate of antifungal use for febrile neutropenia lowering the exposure to expensive and potentially toxic drugs [13–15]. The lack of a standard definition for pre-emptive approach, the variability of data and the possible excess of mortality with this strategy, lead different published guidelines not to grading a recommendation for this approach [11,12,23].

In this study we aimed to describe the epidemiology and treatment approaches in management of IFIs in a real-life cohort of hematological malignancies during 1719 hospitalization and 221 AFTs.

## Methods

Oncohematological patients consecutively treated with AFT at our Hematology Department between January 2010 and July 2017 were identified in the institutional database, and clinical data, diagnostic work-up, treatment modalities, and outcomes were extracted.

We routinely collect microbiological data from hospitalized patients, in particular nasal and rectal swabs are obtained once weekly; in the event of fever (temperature >38°C recorded twice in 1 hour or >38.5°C recorded once), a baseline diagnostic work-up based on two blood cultures (from both central venous catheter and peripheral vein), and other microbiologic (swabs, sputum, urine, stools cultures), and radiologic exams, if clinically indicated, are performed.

Patients with persistent fever after 72 hours of wide spectrum antibacterial therapy or patients with fever relapsing after 48 hours of defervescence, as well as patients with other clinical findings possibly related to an IFI, undergo an intensive diagnostic work-up that include GM serum detection using the Platelia Aspergillus assay (Bio-Rad Laboratories, Marnes-La-Couquette, France) and CT of the chest or radiological examination of other anatomical sites (sinuses, abdomen, CNS) as indicated by clinical signs.

In patients with radiologic evidence suggestive of IFI and negative GM assay, GM is repeated.

A bronchoalveolar lavage (BAL) is performed when radiology showed a pattern suggestive of infection and no positive microbiological cultures from other sites are available.

Diagnosis of IFI was carried out according to the revised European Organization for Research and Treatment of Cancer/Mycoses Study Group (EORTC/MSG) definitions published in 2008 [16].

According to these definitions, a diagnosis of probable pulmonary invasive aspergillosis (IA) required documentation of one of the following specific radiological findings: dense, well-circumscribed nodular lesion with or without a halo sign or air-crescent sign and cavitary lesion associated to mold isolation from the respiratory tract or positive GM test from serum or respiratory specimens [BAL or sputum]. Two consecutive positive serum samples with an index ≥0.5 or a single positive serum sample with an index ≥0.7, or a positive respiratory sample with an index ≥1 were required for a diagnosis of probable IA. A diagnosis of possible IFI was made when the above specific radiological findings were present in the absence of any microbiological documentation.

The choice of first AFT was left to the investigator choice, on the basis of drug indications, diagnostic work-up results and degree of recommendation proposed by the various guidelines [16,18,23]. At the time of drug prescription, in case of suspected invasive aspergillosis [18], voriconazole was the first choice. Liposomal amphotericin B (LAMB) or caspofungin were chosen, started after 72 hours of fever unresponsive to broad-spectrum antibiotic treatment, in case of suspected radiological findings (if not fulfilling EORTC/MSG criteria for IA) [16]. In case of fungal isolates, AFT was driven by antifungal susceptibility, choosing drugs which exhibited the more favorable inhibitory concentrations (MICs).

Unsuccessful outcome was defined as patient death during hospitalization. Attributable mortality was considered as death of a patient with documented radiological, microbiological, histological or clinical findings suggestive of active IFI with no response to treatment when other potential causes of death could be excluded.

The study was approved by ethic committees of Fondazione IRCCS Ca’ Granda—Ospedale Maggiore Policlinico di Milano. Informed written consent was obtained from participants to the study or from family members in case of death.

All procedures followed were in accordance with the ethical standards of the responsible committee on human experimentation (institutional and national) and with the Helsinki Declaration of 1975, as revised in 2008.

### Statistical analyses

Averaged data were expressed as median (range). Chi-square test was used to investigate correlations between all the nominal variables described. Values of *p*<0.05 were considered significant; p NS was used to indicate a statistical non-significance. Analyses were performed using STAT VIEW SAS V. 5.0.

The following nominal variables were analyzed: BAL positive/negative, mycotic isolates (none, yeast, mold), anatomical site of mycotic infection (lung, paranasal sinuses, blood, oropharynx), hematologic diagnosis, galattomannan positive/negative, neutropenia present/absent, previous azole prophylaxis, lung and/or paranasal sinuses CT scan positive/negative, antifungal agents (LAMB, voriconazole, caspofungin), first agent used, type of the sequence used, use of combination AFT, type of combination AFT, EORTC/MSG classification, outcome (alive/dead during hospitalization) [16].

## Results

From January 2010 to July 2017, among 1719 consecutive hospitalizations, 221 AFTs in 196 patients were recorded.

All the patients had a diagnosis of hematological malignancy and persistent fever after 72 hours of antibacterial therapy or fever relapsing after 48 hours of defervescence.

### Clinical characteristics and diagnostic procedures

Clinical details of the studied cohort are listed in **Table 1**.

**Table 1:** Demographic and clinical parameters for the patients who received antifungal therapy.

	Antifungal therapy n. 221
**Age (years), median (range), +/- SD**	61 (18–85) ± 15
**Male/female**	129/92
Hematological malignancies, n (%)*	
** Lymphoma**	48 (22)
** Acute myeloid leukemia**	104 (47)
** Acute lymphoblastic leukemia** ** Chronic lymphocytic leukemia**	20 (9) 14 (6)
** Multiple myeloma** ** Other oncohematological diseases**	10 (5) 25 (11)
Neutropenia, n (%)* (absolute neutrophil count<0.5x10^**9**^**/L)**	177 (80)
Proven IFI, n (%)*	22 (10)
Probable IFI, n (%)*	61 (28)
Possible IFI, n (%)*	81 (37)
Not otherwise classifiable, n (%)*	57 (26)

* Percentage (%) was calculated on total number of AFTs (n. 221), approximated to the nearest whole number.

The most represented disease was AML (104 patients). Median age at the beginning of AFT was 61 years (18–85) and male/female ratio was 129/92 (58/42%). At the fever onset 177 (80%) patients had a neutrophil count <0.5x10^9^/L. Neutropenia frequency was significantly different among the various diseases: 38/48 (79%) lymphoma cases, 17/20 (85%) acute lymphoblastic leukemia (ALL) cases, 95/104 (91%) AML cases, 9/14 (64%) chronic lymphocytic leukemia and 5/10 (50%) multiple myeloma, 13/25 (52%) others diagnoses (p = 0.001, chi square test).

Twenty-nine (13%) patients were receiving antifungal prophylaxis (26 posaconazole, 2 fluconazole, 1 itraconazole). AML was the most frequently disease associated to an antifungal prophylaxis (27/104, 26%, p<0.0001, chi square test).

In one-hundred-ninety-one of 221 (86%) cases a chest (184) and/or sinus (36) CT scans were performed: 163/191 (85%) showed alterations suggestive for IFI. A negative chest CT scan was found in 28/191 (15%) patients. Due to hepatic impairment, 2 patients underwent abdomen CT scan and hepatosplenic mycoses were suspected.

Considering hematological diagnosis, 76/94 (81%) of AML, 14/18 (78%) of ALL and 36/39 (92%) of lymphoma patients who performed a CT scan had a positive result. All patients affected by multiple myeloma presented a positive CT scan (10/10), while 27/30 (90%) of the remaining diseases had a positive CT scan.

Chest CT scan positivity correlated with voriconazole use (82 voriconazole/155, 53%, vs 4 voriconazole/29 negative CT scan, 14%, p<0.0001, chi square test), but not with LAMB or caspofungin treatment. Moreover a chest CT scan positivity correlated with AFT switch (68 switches/155 positive CT scan, 44%, vs 5 switches/29 negative CT scan, 17%, p 0.0071, chi square test) and BAL positivity (45 BAL performed, 42 cases in which BAL and lung CT scan were available, 27 positive BAL/41 positive CT scan, 66%, vs 0 positive BAL/1 negative CT scan, p = 0.0217, chi square test).

The serum GM was evaluated in 203/221 (92%) cases. It was positive in 41/203 (20%) of the tested patients; GM positivity correlated with voriconazole use (33 voriconazole/41 positive GM, 80%, vs 58 voriconazole/162 negative GM, 36%, p<0.0001, chi square test), while GM negativity was significantly associated with the use of LAMB or caspofungin as first agents (70 LAMB, 62 caspofungin and 25 voriconazole treatments out of 162 negative GM cases, p = 0.0367, chi square test).

Considering cases who performed a chest CT scan and a GM test (169), CT scan and GM antigen were contemporarily positive in 36/169 (21%) cases, while a single patient with a CT scan negative for IFI had a positive GM antigen (p = 0.0156, chi-square test). So, if the patient had a positive GM, in 36/37 cases the CT scan was suggestive for IFI (97%).

Having performed an antifungal prophylaxis was not significantly associated with serum GM or CT scan findings, and no statistical difference was found in CT scan results based on the presence of neutropenia (p NS; chi-square test).

BAL was performed in 45/221 cases (20%) and resulted positive for fungal isolates in 30 cases (67%). BAL positivity correlated with the use of voriconazole (20 voriconazole/28 positive BAL, 71%, vs 10 voriconazole/17 negative BAL, 59%, p = 0.0063, chi square test), but not with LAMB or caspofungin use. It did not affect the first agent choice, but it was correlated with the use of combination therapy (p = 0.0383, chi square test): of 18 combination therapies, 6 were performed in 30 positive BALs (20%), 1 in 15 negative BALs (7%) and 11 in 176 cases in which BAL was not performed (6%).

Across the different diagnoses no significant differences in antifungal drug treatment, mycotic isolates, CT scan, GM positivity, BAL were evidenced, suggesting that the clinical pictures were similar and were therefore approached similarly.

### Etiological agents

Sixty-five out of 196 patients (33%) showed positive culture results of samples from different tissues. Candida spp. were identified in 45 isolates, while Aspergillus spp. in 16 cases, as shown in detail in **Table 2**. Fourteen patients presented multiple positivity. In only one case blood cultures were positive for a mold (Fusarium spp), in all other cases molds were isolated from the respiratory tract (BAL, sputum culture or nasal swab), except for one diagnosis of hepatic mycoses made through liver biopsy. Yeasts were isolated from peripheral blood (10 cases) and respiratory tract in most of the remaining cases.

**Table 2:** Fungal agents isolated from oncohematological patients.

Aetiological agent	n. isolates	Site of isolation (n)
**C. glabrata**	14	pharyngeal swab (4), sputum (4), blood (2), BAL (3), coproculture (1)
**C. albicans**	17	pharyngeal swab (6), sputum (6), BAL (3), blood (2)
**C. tropicalis**	5	pharyngeal swab (1), blood (2), sputum (2)
**C. parapsilosis**	3	pharyngeal swab (1), sputum (2)
**C. krusei**	5	pharyngeal swab (3), BAL (1), blood (1)
**C. kefyr**	1	blood (1)
**A. fumigatus**	8	nasal swab (1), sputum (4), BAL (3)
**A. flavus**	5	nasal swab (2), sputum (2), BAL (1)
**A. niger**	3	BAL (3)
**Fusarium spp.**	3	BAL (1), blood (1), nasal swab (1)
**Rizhomucor**	1	BAL (1)
**P. jirovecii**	4	BAL (4)
**Geotrichum capitatum**	5	blood (5)
**Rhodotorula mucilaginosa**	1	blood (1)
**Others**	4	BAL (2), blood (1), liver (1)

BAL: bronchoalveolar lavage.

The GM antigen was positive in 15/65 (23%) patients who had an isolated causative pathogen, while BAL isolates were identified in 23 of the 45 (51%) patients who underwent this procedure.

### EORTC/MSG classification

Based on the results previously exposed, the 221 recorded episodes could be classified according to EORTC/MSG criteria [16] as follows: 22/221 (10%) proven, 61/221 (28%) probable and 81/221 (37%) possible IFIs; 57/221 (26%) patients could not be classified because they did not fulfilled completely the EORTC/MSG criteria. EORTC/MSG classification was not significantly associated with oncohematological diagnosis, antifungal agent used or previous antifungal prophylaxis. Neutropenia presented a significantly different distribution in the various EORT/MSG groups: 20/22 proven cases (91%), 43/61 probable cases (70%), 77/81 possible cases (95%), and 37/57 unclassifiable cases (65%) (p<0.0001 chi square test).

### Antifungal treatments

One-hundred-thirty-one (59%) patients were treated with a single antifungal agent, 81 (37%) with a multiple sequential therapy, while 18 (9 of which after a previous therapy) (8%) patients received a combination regimen. According to hematological diagnosis, 50% of AML (51 cases), 55% of ALL (11 cases), 33% of lymphoma (16 cases), 50% of multiple myeloma (5 cases), 36% of patients with other hematological disease (9 cases) needed more than one drug.

LAMB was the most used antifungal agent (123 cases), followed by caspofungin (100 cases) and voriconazole (93 cases). Among the latter group, 8 patients received voriconazole only at discharge in order to complete the antifungal treatment. Furthermore, LAMB was the most common first antifungal agent used in patients who received antifungal prophylaxis (19/29, 66%, p = 0.0359, chi-square test).

Eighty-one patients received a sequential therapy; the most used sequences were LAMB and voriconazole (20 cases), LAMB and caspofungin (17 cases), voriconazole and caspofungin (13 cases), amphotericin and caspofungin (4 cases). The most frequent used combination therapies were as follows: LAMB plus voriconazole (6 cases), caspofungin plus LAMB (6 cases), and caspofungin plus voriconazole (4 cases).

The presence of a mycotic isolate significantly correlated with voriconazole (37 voriconazole/65 positive isolates, 57%, vs 56 voriconazole/156 negative isolates, 36%) or caspofungin use (39 caspofungin/65 positive isolates, 60%, vs 61 caspofungin/156 negative isolates, 39%) (p<0.0001 and p = 0.0004, chi square test, respectively). In the group of 50 cases without mycotic isolates undergoing antifungal drug switch, the most frequent sequences were LAMB-voriconazole (16 cases, 32%) and caspofungin-voriconazole (13 cases, 26%) (p<0.0001, chi square test). Moreover, the presence of a mycotic isolate correlated significantly with the use of association therapy (15 association AFT/65 positive isolates, 23%, vs 3 association AFT/156 negative isolates, 2%, p<0.0001, chi square test).

In the small group of 29 patients that were exposed to azole-based prophylaxis, only 6 (21%) were treated at any time with voriconazole, compared to 87/192 treatments (45%) in the azole naïve cases (p = 0.0123, chi square test). Conversely, 24/29 (83%) cases exposed to azole prophylaxis and 99/192 (52%) azole naïve cases were treated with LAMB at any time (p = 0.0016, chi square test). Consistently, the first agent used after azole prophylaxis was LAMB (19/29, 66%), followed by 8/29 (28%) cases of caspofungin and 1/29 (3%) cases of voriconazole or fluconazole (p = 0.0359, chi square test).

Antifungal susceptibility tests were available for 13/22 proven IFI. On the basis of antibiograms an adequate AFT was administered in all proven cases, except for one patient with a C. krusei fungemia who started voriconazole therapy then switched to caspofungin plus LAMB according to sensitivity, and a Rhodotorula mucilaginosa fungemia initially treated sequentially with caspofungin and LAMB. All details about antifungal susceptibility was shown in **Table 3**.

**Table 3:** Available antifungal susceptibility of fungal isolates from patients with hematologic malignances.

Species (n)	MIC (mg/L)				Performed therapy	Outcome
	Fluconazole	Voriconazole	LAMB	Caspofungin		
Candida glabrata (1)	128	R (NA)	1	S (NA)	A, C	positive
Candida albicans (3)	0,5 1 0,5	<0,008 0,06 0,015	0,5 1 0,5	0,06 0,06 0,03	C, F C A+C	negative negative negative
Candida krusei (1)	128	2	1	0,5	V, A+C	negative
Geotrichum capitatum (4)	8 4 R (NA) 32	0,12 0,06 S (NA) 0,5	0,5 1 S (NA) 1	0,5 R (NA) R (NA) R (NA)	A+V A A, V A+V	positive positive positive negative
Rhodotorula mucillaginosa (1)	256	8	1	8	C, A	positive
Aspergillus flavus (1)	NA	0,19	0,019	NA	V, A+C	negative
Aspergillus fumigatus (1)	NA	0,06	0,38	NA	V	positive
Fusarium spp (1)	NA	0,75	1,5	NA	A, V	positive

MIC: minimum inhibitory concentration; R: resistant; S: susceptible; NA: MIC number not available; A: ambisome; C: caspofungin; V: voriconazole; F: fluconazole; +: in combination.

### Outcome

Forty-one patients died during the follow-up period. The cause of death was attributed to IFI in all 41 cases. Twenty-five patients had a diagnosis of AML, 1 of ALL, 7 of chronic lymphocytic leukemia/lymphoma, 2 MM and 6 were in the category other malignancies (**Table 4**). AML diagnosis correlated with a poor outcome (p = 0.04, chi square test) when compared to all the other diagnoses.

**Table 4:** Outcome of patients treated with antifungal drugs.

	Positive	Negative	p (chi-square test)
**AML, n (%)**	79 (76%)	25 (24%)	0.04
**Positive CT, n (%)**	133 (82%)	30 (18%)	
**Negative CT, n (%)**	25 (89%)	3 (11%)	0.32
**CT not done, n (%)**	22 (73%)	8 (27%)	
**Positive GM antigen, n (%)**	33 (80%)	8 (20%)	
**Negative GM antigen, n (%)**	133 (82%)	29 (18%)	0.89
**GM antigen not done, n (%)**	14 (78%)	4 (22%)	
**Proven IFI, n (%)**	15 (68%)	7 (32%)	
**Probable IFI, n (%)**	48 (79%)	13 (21%)	
**Possible IFI, n (%)**	69 (85%)	12 (15%)	0.27
**Not classifiable, n (%)**	48 (84%)	9 (16%)	
**Monotherapy, n (%)**	109 (83%)	22 (17%)	
**Sequential or association therapy, n (%)**	66 (81%)	15 (19%)	0.75

Percentage (%) was calculated on total number of AFTs for each listed category.

According to EORTC/MSG [16] criteria IFI could be classified as proven in 7, probable in 13, possible in 12 and unclassifiable in 9 cases.

No correlations were found between outcome during hospitalization (positive outcome, alive/negative outcome, dead) and the following variables (see Methods section): BAL pos/neg, mycotic isolates (none, yeast, mold), anatomical site of mycotic infection (lung, paranasal sinuses, blood, oropharynx), galattomannan pos/neg, neutropenia present/absent, previous azole-prophylaxis, lung and paranasal sinuses CT scan positivity/negativity, first antifungal agent used, use of combination antimycotic therapy, type of combination therapy, EORTC/MSG classification [16].

Voriconazole use was more frequent (82 voriconazole/180 positive outcomes, 46%, vs 11 voriconazole/41 negative outcomes, 27%, p = 0.0252, chi square test) in patients with a positive outcome, caspofungin use was more frequent in patients with a negative outcome (73 caspofungin/180 positive outcomes, 41%, vs 27 caspofungin/41 negative outcomes, 66%, p = 0.030, chi square test), while LAMB use was equally represented in the two groups.

Among patients with a poor outcome, GM serum was positive in 8/37 (22%) tested patients. Among patients with a positive outcome, GM serum was positive in 33/166 (20%) tested patients. Within proven IFI for what an antifungal susceptibility test was available, two patients were exposed to an initial inadequate AFT: of these 1 died of a C. krusei fungemia (1/41 negative outcome, 2%), while the other case had a positive outcome (1/179 positive outcome, 0.6%, p NS, chi square test).

**Fig 1:**
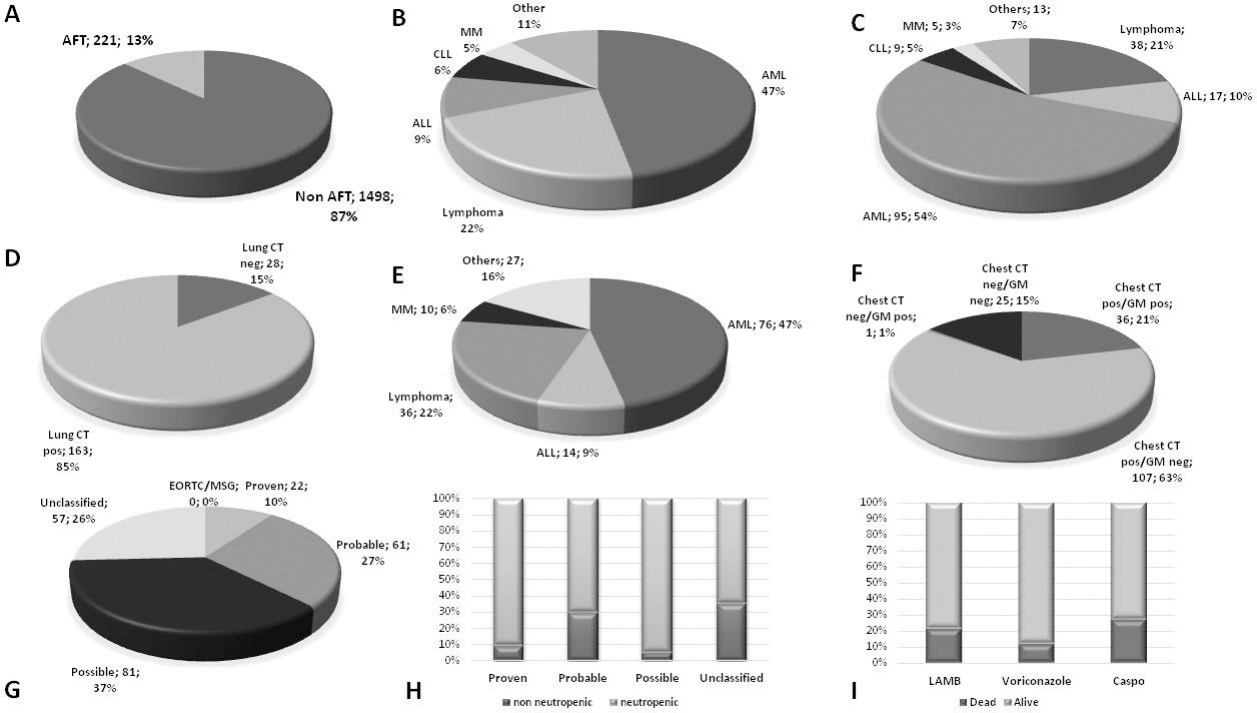
Clinical characteristics, diagnostic procedures, EORTC/MSG classification, distribution of AFT and association with outcome. (A) Proportion of patients undergoing or not undergoing to AFT. (B) Diagnoses among the group of patients undergoing AFT. (C) Distribution of neutropenia in the patients undergoing to AFT. (D) Proportion of lung CT scans positive and negative in the patients undergoing AFT. (E) Distribution of positive lung CT scan across the different diagnoses. (F) Distribution of GM and lung CT scan positivity/negativity. (G) EORTC/MSG classification of the AFT patients. (H) Distribution of neutropenia in the different EORTC/MSG classes. (I) Distribution of different antifungal agents among positive (alive) and negative (dead) outcomes.

**Fig 1** shows a summary of clinical characteristics, diagnostic procedures, EORTC/MSG classification, distribution of AFT and association of these features with the outcome.

## Discussion

IFI is a prominent cause of morbidity and mortality in patients with hematological malignancies and HSCT recipients [1–3]. In the present retrospective study, we report the results of a single center real-life experience in a large series of 1719 hospitalization and 221 AFTs in 196 patients with oncohematological diseases. We confirm the relevance of EORTC/MSG criteria in the clinical practice: host factors, CT, GM and microbiological isolates, which characterized our series, and guided the therapeutic choices.

In particular, in the AFT treated population, AML diagnoses predominates (47%). Consistently, in the Hema-e-Chart Registry, >80% of IFI was represented by AML patients [4], while in the SEIFEM-2004 study this percentage was 69% [1], confirming the ‘high risk’ of IFI in this set of patients. In our series, other hematological malignancies were less represented, but it is interesting to note that also lymphoma and ALL patients presented a rate of neutropenia similar to AML, confirming the well known relevance of neutropenic phase in the risk of contracting IFIs.

According to the literature, for three consecutive positive serum GM tests ≥0.5, sensitivity/specificity was 73.3%/85.9% with an accuracy of 84% [20]. These values seems to be more reliable in the case of angioinvasive aspergillosis rather than in airway invasive cases. Furthermore, some antifungals or antibiotics may influence the GM testing sensitivity, and false-positive or false-negative results are sometimes obtained [18,20]. In our cohort, the serum GM was positive in 20% of the tested patients, but when positive, it correlated with a CT scan suggestive for IFI in 97% of the cases, indicating that the combined use of GM and chest CT scans may promote a timely diagnosis of invasive aspergillosis in oncohematologic patients with febrile neutropenia [18,21].

New tests (e.g. 1-3-β-D-glucan determination) have still to demonstrate their reliability [19]; the diagnostic strength of any new marker is usually measured referring to the EORTC/MSG IFI diagnostic criteria [16], however, it is clear that the only group in which the sensitivity and specificity of a new test may be reliably allocated is the rare group of proven IFIs, and this represents an objective theoretical difficulty to be taken into account in the validation of new IFI markers.

In the present study, the main anatomical site targeted by mold IFI remained the lung, as diagnosed by CT scan, whereas disseminated mold infection was a rare event, occurring in one case of Fusarium fungemia. On the other hand, yeasts were isolated much more frequently from peripheral blood. In our series, Candida spp. represented the main etiological agent identified, followed by Aspergillus spp. We confirmed the relevant incidence of non-albicans Candida spp and non-fumigatus Aspergillus spp., as reported in other recent studies [1–7]. Compared to our findings, SEIFEM-2004 study and Hema-e-Chart registry reported a higher incidence of aspergillosis [1,4]. In the SEIFEM-2004 study over half IFIs (346/538) were caused by molds, in most cases Aspergillus spp. (310/346), while the 192 yeast infections included 175 cases of candidemia [1]. In the Hema-e-Chart Registry a yeast/mold ratio of 1/2.2 was reported [4]. Nevertheless, in both studies, if only proven infection were considered, yeasts represented the largest group, mainly due to fungemia occurrence [1,4].

According to EORTC/MSG IFIs diagnostic criteria [16], we were able to classify 83 (38%) cases as proven/probable IFIs. In the other cases, antimycotic drugs were administered in IFIs graded as possible (38%) or, in the unclassifiable group (26%), according to an empiric approach. The high frequency of cases unclassifiable by EORTC/MSG criteria [16] represents a point of attention and may be interpreted in at least two different ways: it can be expression of an overtreatment (in other words the patients were not affected by IFI and received AFT inappropriately) or, conversely, diagnostic criteria were not sufficiently extensive to identified all the cases of IFIs.

Therefore, a careful evaluation of oncohematologic patients with suspected IFI must be carried out. In fact, empiric therapy, more properly defined fever-driven, may result in the inappropriate administration of toxic and expensive drugs. Nevertheless, this approach is potentially associated to a reduced mortality in oncohematologic patients receiving an early AFT, compared to a pre-emptive (or diagnosis-driven) approach, characterised by a longer time to treatment [12–15]. Furthermore, in hematological malignancies, the appearance of infection/inflammation signs, in particular typical lung nodules, might by blunted by the profound state of immunosuppression, with the risk of delaying treatment when diagnosis-driven (pre-emptive) approach is rigorously applied. Partially supporting this hypothesis, we observed a significant group (from 10 to 20%) of patients treated with AFT showing a negative CT scan: in these cases, the profound immune suppression might have contributed to reducing inflammatory responses and therefore radiological evidence.

Nevertheless, we confirm the central role of CT scan in the management of suspected IFIs, as supported by the high proportion of cases who performed it, the high number of positive CT scans and its relevance in the choice of antifungal treatment, suggested by the correlation between CT positivity and the class switch and also the use of LAMB and voriconazole.

In our study, the most commonly used antifungal agent was LAMB, followed by caspofungin and voriconazole. LAMB was also the most common first antifungal agent used in patients who received antimycotic prophylaxis.

Focusing on the small patient group that received azole-based prophylaxis, the vast majority of these cases were treated with LAMB at any time, and, consistently, the most frequent first agent used after azole prophylaxis was LAMB.

The reason for these therapeutic choices is coherent with antifungal class switch in patients who underwent to a mold active prophylaxis, as suggested by the published guidelines [18].

Fifty-nine percent of the patients received single antifungal agent, 37% a sequential therapy and 8% a combination regimen. The presence of a mycotic isolate correlated significantly with the use of association therapy, and a chest CT scan or a GM positivity correlated with AFT switch. Moreover, BAL positivity was correlated with the use of combination therapy. These data underlined the prevailing use of combination regimen or antifungal switch in patients with a proven/probable IFI, rather than a fungal infection classified as possible.

These observations prompt the following considerations: BAL is performed in almost one- third of positive lung CT scan, a proportion that should be increased in order to maximize the possibility of a better oriented therapy, as it allows isolation of a fungal agent in approximately two-thirds of cases. Furthermore its relevance to AFT choice, is suggested by its association with voriconazole use and with the combination therapy.

Our results indicated that the rate of systemic AFT, the incidence of probable/proven IFI and IFI-attributable mortality were 13%, 5% and 20%, respectively, which were comparable with the findings reported from other studies in this particular setting [1–4,24,25]. The SEIFEM-2004 study reported an incidence of IFI of 4.6%, and IFI-attributable mortality rate was 39%, higher than ours [1]. In two recent studies from Turkish and Taiwan groups incidence of probable/proven IFI was 6.7% and 5.6% and IFI-attributable mortality was 14.2% and 5.9% respectively [24,25]. In the Hema-e-Chart registry 14% of patients died within 12 weeks after the start of antifungal therapy; IFI-attributable mortality was 30.4% and 17.3% in yeast and proven/probable mold infections, respectively [4].

When we consider specifically studies focused on candidemia, the SEIFEM 2015-B report showed among 16,529 patients treated with conventional chemotherapy a total of 135 candidemia for an overall incidence of 0.8% and a case fatality rate of 22%, while for invasive aspergillosis the SEIFEM-2008 study demonstrated a mortality rate attributable of 27% on 140 of proven/probable cases [26,27].

These data indicate that mortality rates remain high, although the incidence of IFI has declined in the past decade due to multiple reasons, among which are certainly anti-mold prophylaxis and early treatment implemented in the clinical practice [1–7].

In our series, voriconazole use was associated with a positive outcome, while caspofungin use was associated with a negative outcome. The reason is not clear, but we speculated that it might be associated with higher mortality from yeast compared with mold infections.

In conclusion, we described a large series of AFT in oncohematologic patients. IFIs and AFT frequency remains relevant, as well as mortality, in spite of the application of very aggressive diagnostic procedures and timely administration of AFT.

In addition, we confirmed the clinical relevance for AFT choice of multi-step diagnostic approach composed of CT scan, BAL, GM and microbiological results. Furthermore, the still high proportion of cases not classifiable with EORTC/MSG criteria, indicate that IFI diagnosis still represent a field were active research is needed to further improve diagnostic accuracy, with potentially very relevant therapeutic implications.
